# Improving Optical Flow Sensor Using a Gimbal for Quadrotor Navigation in GPS-Denied Environment

**DOI:** 10.3390/s24072183

**Published:** 2024-03-28

**Authors:** Jonathan Flores, Ivan Gonzalez-Hernandez, Sergio Salazar, Rogelio Lozano, Christian Reyes

**Affiliations:** Department of Research and Multidisciplinary Studies, Program of Aerial and Submarine Autonomous Navigation Systems, Center for Research and Advanced Studies of the National Polytechnic Institute, Mexico City 07360, Mexico; jonathan.flores@cinvestav.mx (J.F.); ivan.gonzalez@cinvestav.mx (I.G.-H.); sesalazar@cinvestav.mx (S.S.); christian.reyes@cinvestav.mx (C.R.)

**Keywords:** UAV, navigation, robust control, GPS-denied

## Abstract

This paper proposes a new sensor using optical flow to stabilize a quadrotor when a GPS signal is not available. Normally, optical flow varies with the attitude of the aerial vehicle. This produces positive feedback on the attitude control that destabilizes the orientation of the vehicle. To avoid this, we propose a novel sensor using an optical flow camera with a 6DoF IMU (Inertial Measurement Unit) mounted on a two-axis anti-shake stabilizer mobile aerial gimbal. We also propose a robust algorithm based on Sliding Mode Control for stabilizing the optical flow sensor downwards independently of the aerial vehicle attitude. This method improves the estimation of the position and velocity of the quadrotor. We present experimental results to show the performance of the proposed sensor and algorithms.

## 1. Introduction

Research around Unmanned Aerial Vehicles (UAVs) has increased in recent decades due to their capacity for experimentation in different disciplines, low cost, and ease of repair compared to classical helicopter configurations. Sensor improvements and robust control algorithm developments allow for accuracy tasks in vehicles of different configurations such as fixed-wing aircrafts, rotorcrafts, and hybrids. Sensor-based navigation approaches are developed in specific environments and conditions for civil, military, or scientific applications.

In the early stages of development, optical flow-based navigation was limited by the range and field of view of the sensors. The frame-fixed optical flow module uses a downward-facing camera and a distance sensor for velocity estimation. Optical flow detection is affected due to angle changes caused by UAV maneuvers. In hover flights the maneuvers are reduced, and the velocity estimation is reliable.

Motion capture can be used for high-precision indoor flight. In [1], the position of the multirotor aircraft is estimated by a set of cameras in a room; the tracking of the movement of the UAV was controlled offline to follow a ground vehicle, but the cost of the motion capture system is highlighted. The works [2,3] present open-air flights using GPS in precise long-range missions, analyze the sensitivity of GPS to interference, and propose a return-to-home flight mode using the heading angle to home. In [4], vision-based navigation is used for indoor and outdoor flights with high-cost computational requirements and lighting changes sensitivity. Moreover, navigation using optical flow and distance sensors requires low-cost computational and provides good accuracy in local environments.

In [5], the authors present an eight-rotor UAV using optical flow, and this rotorcraft uses the main four rotors for altitude and attitude displacement and another four rotors for horizontal maneuvers. This prototype uses the optical flow for estimating the horizontal position avoiding angular tilt. In [6] in the authors proposed improved motion compensation using feature block selection, look-ahead rotation, fault case detection and filtering using PX4FLOW hardware by tunning the camera resolution, the interval of adaptive boxes and advanced search algorithm. In [7] presents a UAV position estimation using an optical flow approach using a Gated Recurrent Unit (GRU) network-based pointing angles and Magnetic, Angular Rate, and Gravity (MARG) sensors improving robustness and performance in real-time experimentation.

Optical flow sensors used for position control in quadrotor aircraft are facing downwards and fixed to the airframe. In a flight based on the optical flow sensor, the roll and pitch maneuvers are restricted to $\pm 15^{\circ }$, while maneuvers in flights without optical flow sensors are up to $\pm 35^{\circ }$. The bounded angles reduce the horizontal displacement velocity of the vehicle. Quadrotor rotorcraft in an ideal hover flight would maintain the Euler angles of pitch and roll aligned to the horizontal and with a linear displacement velocity near zero. However, in practice (even in hover flight), a quadrotor rotorcraft vehicle is sensitive to disturbances and the responses of the attitude controller can be considered as optical noise. Furthermore, it is well known that tilting an optical flow module produces positive feedback on vertical position estimates and linear velocity estimates.

**Figure 1:**
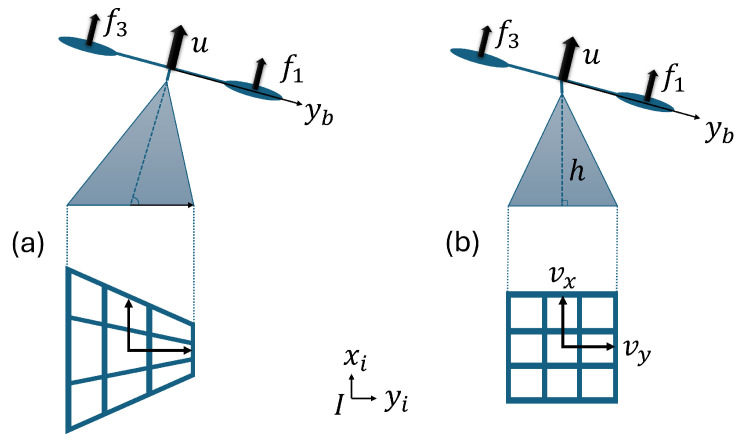
(**a**) Optical flow without stabilization and (**b**) stabilized optical flow.

The rotorcraft in Figure 1a shows an optical flow module mounted directly on the quadrotor’s frame. When the quadrotor tilts it causes a distortion in the estimation of the altitude and horizontal velocity. Figure 1b shows an optical flow module stabilized by a gimbal. The gimbal feedback control stabilizes pitch and roll angles on the horizontal plane, providing reliable altitude and horizontal velocity estimations with respect to the ground and avoiding optical distortions.

The main contribution of the present paper is improving the optical flow sensing to obtain a reliable set of image data using a gimbal that in general is used to stabilize camera videos for recording or First-Person View (FPV) flights. The optical flow sensor is improved for trajectory control in GPS-Denied environments. This approach allows for flight maneuvers with large changes in the attitude angles; in other cases this produces positive feedback that destabilizes the orientation of the vehicle. Furthermore, we use a robust control technique to enable hover flights and trajectory following with tracking velocity without affecting the sensed images.

The paper is organized as follows: Section 2 describes the system mathematical models. Section 3 is devoted to the control algorithms. Materials and Methods are given in Section 4. The experimental results are shown in Section 5. A brief discussion about our approach is presented in Section 6. Final remarks are given in the conclusions.

## 2. Mathematical Models

Mathematical models are approximations of the real systems. The subsystems include the dynamics, estimations, control inputs, and degrees of freedom. In the quadrotor aircraft, the forces and angular moments are induced by four actuators to move in the inertial reference frame (x,y,z) and rotate in their Euler angles (ϕ,θ,ψ). In the gimbal, the angular moments are induced by its three actuators that allow it to tilt around its Euler angles $\left(\phi_{g},\theta_{g},\psi_{g}\right)$.

The optical flow module estimates the lateral and longitudinal displacement velocity and altitude of the complete system $\left(\dot{x},\dot{y},z\right)$. The following subsections present the mathematical models of the quadrotor aircraft, gimbal, and optical flow module to describe the complete system.

### 2.1. Quadrotor Aircraft Model

**Figure 2:**
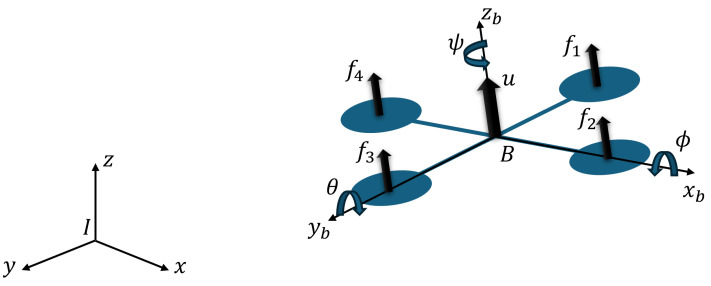
Quadrotor aircraft diagram.

Figure 2 shows the representation of the quadrotor aircraft. Let us define the total thrust *u* as follows:

(1) $$u=(f_{1}+f_{2}+f_{3}+f_{4})$$

where $f_{i}=k\omega_{i}^{2}$ is the force produced by *i*-rotor where i=1,2,3,4. *k* represents a set of aerodynamic constants, and $\omega_{i}$ is the angular velocity. We obtain the Euler angles rates as

$$\left[\begin{array}{c} \dot{\phi }\\ \dot{\theta }\\ \dot{\psi } \end{array}\right]=T\left[\begin{array}{c} p\\ q\\ r \end{array}\right]$$

where

$$T=\left[\begin{array}{ccc} 1 & \sin\phi \tan\theta & \cos\phi \tan\theta \\ 0 & \cos\phi & -\sin\phi \\ 0 & \sin\phi \sec\theta & \cos\phi \sec\theta \end{array}\right]$$

The rotor torque $\tau_{\left(\phi ,\theta ,\psi \right)}$ is defined as

(2) $$\begin{array}{cc} \tau_{\phi }= & (f_{1}-f_{3})l\\ \tau_{\theta }= & (f_{2}-f_{4})l\\ \tau_{\psi }= & \sum_{i=1}^{4}\tau_{i} \end{array}$$

where *l* is the distance from the rotors to the center of the gravity and $\tau_{i}$ is the torque produced by the *i*-rotor. Therefore, according to [8] the quadrotor aircraft model can be defined as

(3) $$\begin{array}{c} m\ddot{x}=-u\sin\theta \\ m\ddot{y}=u\cos\theta \sin\phi \\ m\ddot{z}=u\cos\theta \cos\phi -mg\\ I\ddot{\eta }+C\left(\eta ,\dot{\eta }\right)\dot{\eta }=\tilde{\tau } \end{array}$$

where *m* is the mass, *g* is the acceleration due to the gravity, *I* represents the inertia matrix, *C* is the Coriolis matrix, $\eta ={\left(\phi ,\theta ,\psi \right)}^{T}$ is the attitude vector, and $\tilde{\tau }$ is the control input vector.

### 2.2. Gimbal Model

**Figure 3:**
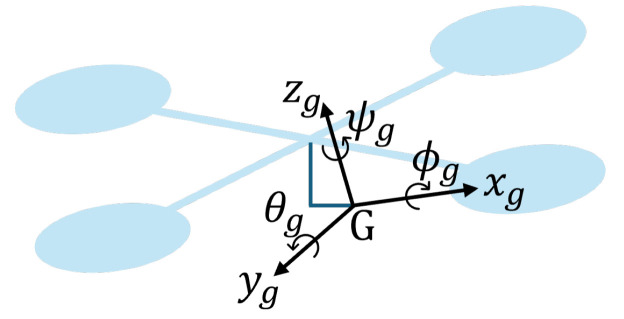
Gimbal diagram.

Figure 3 shows the gimbal diagram, where the $G=(x_{g},y_{g},z_{g})$ represents the frame fixed to the optical flow sensor. In [9,10], the authors propose the gimbal mathematical model as follows:

(4) $$\begin{array}{c} J_{x}{\ddot{\phi }}_{g}+\xi_{1}{\dot{\phi }}_{g}=\upsilon_{\phi }+d_{\phi }\\ J_{y}{\ddot{\theta }}_{g}+\xi_{2}{\dot{\theta }}_{g}=\upsilon_{\theta }+d_{\theta }\\ J_{z}{\ddot{\psi }}_{g}+\xi_{3}{\dot{\psi }}_{g}=\upsilon_{\psi }+d_{\psi } \end{array}$$

where $J=D(J_{x},J_{y},J_{z})$ is the gimbal rotational inertia matrix, and $\eta_{g}=\left(\phi_{g},\theta_{g},\psi_{g}\right)$ represents the torques in the pitch, roll, and yaw gimbal angles. $\xi =(\xi_{1},\xi_{2},\xi_{3})$ are the friction coefficients for each angle. Moreover, $\upsilon =\left(\upsilon_{\phi },\upsilon_{\theta },\upsilon_{\psi }\right)$ is the control input, and $d=\left(d_{\phi },d_{\theta },d_{\psi }\right)$ is the nonlinear dynamic effect of the couplings that are considered as a disturbance.

### 2.3. Optical Flow Model

Optical flow is a monocular vision method that uses images of a moving object to estimate the displacement velocity.

**Figure 4:**
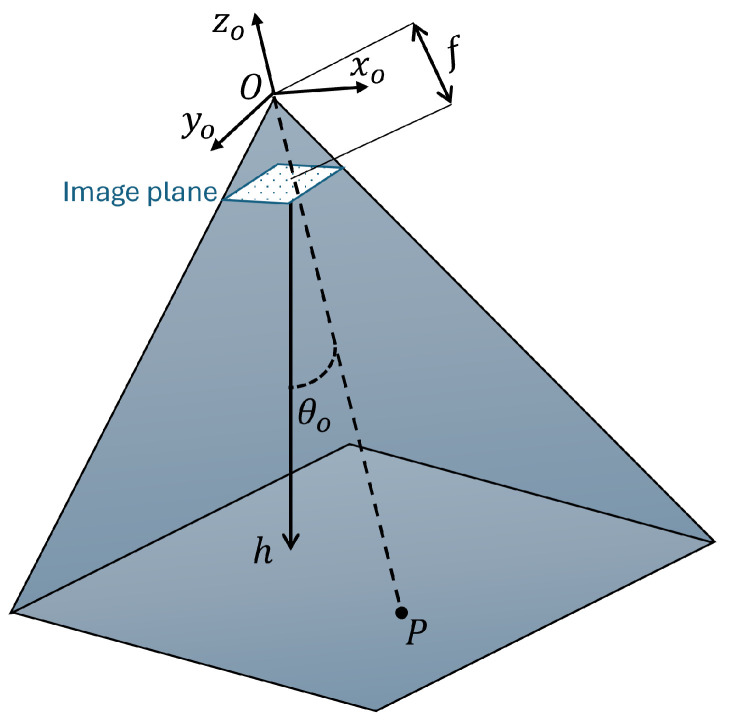
Optical flow diagram.

Figure 4 shows the optical flow diagram, where the $O=(x_{o},y_{o},z_{o})$ represents the frame fixed of the optical flow sensor. *P* is a direct point on the ground from the optical axis, *f* is the focal length from the centre of projection *O* to the image plane, and *h* is the altitude from the image plane to the ground. $\theta_{o}$ is the optical axis angle and the $z_{i}$ axis. The authors of [11] present the approximate pixel displacements as follows:

(5) $$\begin{array}{c} V_{x}=\frac{T_{z}x-T_{x}f}{h}-\omega_{y}f+\omega_{z}y+\frac{\omega_{x}xy-\omega_{y}x^{2}}{f}\\ V_{y}=\frac{T_{z}y-T_{y}f}{h}+\omega_{x}f-\omega_{z}x+\frac{\omega_{x}y^{2}-\omega_{y}xy}{f} \end{array}$$

where $\left(V_{x},V_{y}\right)$ are the estimate optical flow translation velocities, $\left(\omega_{x},\omega_{y},\omega_{z}\right)$ are the angular velocities, and $\left(T_{x},T_{y},T_{z}\right)$ are the linear velocities.

## 3. Control Strategies

This section presents a feedback control algorithm implemented to stabilize the quadrotor aircraft altitude and the robust control algorithms that guarantee finite-time convergence for the quadrotor aircraft pitch and roll angles and for the gimbal pitch and roll angles. The main goal of the algorithms based on Sliding Mode Control is to stabilize the quadrotor aircraft attitude and the optical flow module attitude independently. The quadrotor aircraft altitude and attitude are controlled by the aircraft’s four rotors angular velocity, and the gimbal is controlled by its two rotor torques that stabilize the optical flow module on the horizontal line.

### 3.1. Quadrotor Aircraft Control

The quadrotor aircraft robust control approach enables stable flight by damping external disturbances or attitude tilts for horizontal vehicle displacement. This behavior is very important for the entire aerial vehicle system.

#### 3.1.1. Vertical Displacement and Yaw Control (z,ψ)

A feedback control algorithm has been selected to stabilize the vertical position, and it is obtained using the following input:

(6) $$u=\left(\frac{c_{1}+mg}{\cos\phi \cos\theta }\right)$$

where $c_{1}$ is defined as

(7) $$c_{1}=-k_{p}\left(z-z_{d}\right)-k_{d}\dot{z}$$

where $k_{p},k_{d}>0$ and $z_{d}$ is the desired altitude. Introducing (6) into (3)

(8) $$\ddot{z}=-k_{p}\left(z-z_{d}\right)-k_{d}\dot{z}$$

When the quadrotor aircraft reaches the desired altitude $z\to z_{d}$ then $c_{1}\to 0$.

The yaw angle ψ is controlled by

(9) $$\tau_{\psi }=-k_{a}\left(\psi -\psi_{d}\right)-k_{b}\dot{\psi }$$

where $k_{a},k_{b}>0$ and $\psi_{d}$ means the desired yaw angle.

#### 3.1.2. Lateral Displacement and Roll Control (y,ϕ)

By applying (6) into Equation (3), after a convergence time, the lateral dynamics become

(10) $$\ddot{y}\approx mg\tan\phi$$

In order to guarantee the robust tracking in *y*-axis, differentiating (10) twice gives

(11) $$y^{\left(4\right)}=mg\left(\ddot{\phi }+2\dot{\phi^{2}}\tan\phi \right)\sec^{2}\phi$$

Introducing (3) into (11) and applying a variable change $\tilde{\tau }=\tau +C\left(\eta ,\dot{\eta }\right)\dot{\eta }$ and inertia are considered the identity matrix

(12) $$\begin{array}{cc} y^{\left(4\right)}= & mg\left(\tau_{\phi }+2{\dot{\phi }}^{2}\tan\phi \right)\sec^{2}\phi \\ \;= & \tau_{\phi }mg\sec^{2}\phi +2mg{\dot{\phi }}^{2}\tan\phi \;\sec^{2}\phi \end{array}$$

Let us define the following $f_{a}$ and $f_{b}$ as

(13) $$f_{a}\left(\phi \right)=\left(mg\sec^{2}\phi \right),\;f_{b}\left(\phi ,\dot{\phi }\right)=2mg{\dot{\phi }}^{2}\tan\phi \sec^{2}\phi$$

where $f_{a}\left(\cdot \right)$, $f_{b}\left(\cdot \right)$ represents terms obtained after a second derivative. We have considered

(14) $$f_{a}\left(\phi \right)>0$$

Then, the *y* dynamics are

(15) $$y^{\left(4\right)}=\tau_{\phi }f_{a}\left(\phi \right)+f_{b}\left(\phi ,\dot{\phi }\right)$$

In order to reach the desired trajectory, we propose the robust control based on the Sliding Mode Control algorithm. Let us define the following variables:

(16) $$\begin{array}{cc} {\dot{y}}_{1} & =\dot{y}\\ {\dot{y}}_{2} & =\ddot{y}\\ {\dot{y}}_{3} & =y^{\left(3\right)}\\ {\dot{y}}_{4} & =y^{\left(4\right)}=\tau_{\phi }f_{a}\left(\phi \right)+f_{b}\left(\phi ,\dot{\phi }\right) \end{array}$$

and the following errors as

(17) $$\begin{array}{cc} e_{1} & =y-y_{d}\left(t\right)\\ e_{2} & =\dot{y}-{\dot{y}}_{d}\left(t\right)\\ e_{3} & =\ddot{y}-{\ddot{y}}_{d}\left(t\right)\\ e_{4} & =y^{\left(3\right)}-y_{d}^{\left(3\right)}\left(t\right) \end{array}$$

Differentiating

(18) $$\begin{array}{cc} {\dot{e}}_{1} & =e_{2}\\ {\dot{e}}_{2} & =e_{3}\\ {\dot{e}}_{3} & =e_{4}\\ {\dot{e}}_{4} & =y^{\left(4\right)}-y_{d}^{\left(4\right)}\left(t\right) \end{array}$$

The sliding surface is defined as a function of error

(19) $$s=\left(k_{1}e_{1}+k_{2}e_{2}+k_{3}e_{3}\right)+e_{4}$$

where $k_{1},k_{2},k_{3}>0$. The objective is to reach the surface s=0. We propose the following positive function:

$$V=\frac{1}{2}s^{2}$$

we have

$$\dot{V}=s\left(k_{1}e_{2}+k_{2}e_{3}+k_{3}e_{4}+\tau_{\phi }f_{a}\left(\phi \right)+f_{b}\left(\phi ,\dot{\phi }\right)\right)$$

By choosing $\tau_{\phi }$ like

(20) $$\tau_{\phi }=-\frac{1}{f_{a}\left(\phi \right)}\left[f_{b}\left(\phi ,\dot{\phi }\right)+\left(k_{1}e_{2}+k_{2}e_{3}+k_{3}e_{4}\right)+\nu_{\phi }\right]$$

where $\nu_{\phi }$ is defined as

(21) $$\nu_{\phi }=\rho_{\phi }\;sign\left(s\right)$$

where $\rho_{\phi }>0$ and $\nu_{\phi }$ denote the Sliding Mode Controller, then $\dot{V}=-\rho_{\phi }s\;sign\left(s\right)$. Then, s→0 in a finite time. Then, from (19)

$$e_{4}=-\left(k_{1}e_{1}+k_{2}e_{2}+k_{3}e_{3}\right)$$

this is an error linear system, where the Hurwitz dynamics characteristic polynomial is

$$p^{3}+k_{3}p^{2}+k_{2}p+k_{1}=0$$

then from (18) $e_{1},e_{2},e_{3},e_{4}\to 0$.

#### 3.1.3. Longitudinal Displacement and Pitch Control (x,θ)

After a transient time, the longitudinal dynamics become

(22) $$\ddot{x}\approx -mg\frac{\tan\theta }{\cos\phi }$$

Consider it diagonal and compensate the torque. Considering a small angle ϕ, (22) becomes approximately $\ddot{x}\approx -mg\tan\theta$. Indeed, since the quadrotor aircraft configuration is a symmetrical frame, it is also possible to apply the Sliding Mode Controller to the pitch angle (θ) and to the longitudinal displacement in the *x*-axis.

### 3.2. Gimbal Control

The gimbal robust control approach ensures that the attitude of the optical flow module is stable and independent of the quadrotor aircraft attitude. This performance is very important for optical flow sensing.

#### 3.2.1. Gimbal Pitch Control ($\theta_{g}$)

An algorithm based on Nonsingular Terminal Sliding Mode Control (NTSMC) is selected to stabilize the gimbal pitch angle ($\theta_{g}$) due to the robustness to disturbances.

Since the tracking error converges to zero, we propose the following sliding surface:

(23) $$s_{g}=\theta_{g}+\alpha \;sign^{\gamma_{1}}\left(\theta_{g}\right)+\beta \;sign^{\gamma_{2}}\left({\dot{\theta }}_{g}\right)$$

where $sign^{\gamma }\left(\theta_{g}\right)=sign\theta_{g}{\left|\theta_{g}\right|}^{\gamma }$, $\theta_{g}$ is the gimbal pitch angle, and the variables satisfying α,β>0, $1>\gamma_{1}>2$, $\gamma_{1}>\gamma_{2}$ and sign(·) are the signum function. Then, $s_{g}$ guarantees the convergence to zero in finite time. The algorithm control law $\upsilon_{\theta }$ is defined as

(24) $$\upsilon_{\theta }=-J_{y}\left[\frac{1}{\beta \gamma_{2}}sign^{2-\gamma_{2}}{\dot{\theta }}_{g}\left(1+\alpha \gamma_{1}{\left|\theta_{g}\right|}^{\gamma -1}\right)+\beta {\dot{\theta }}_{g}+K_{1}sign\left(s\right)+K_{2}s\right]$$

where $K_{1},K_{2}>0$. The control law $\upsilon_{\theta }$ for the pitch ($\theta_{g}$) angle guarantees the finite-time convergence of the tracking error to zero. The stability proof can be found in [12].

#### 3.2.2. Gimbal Roll Control ($\phi_{g}$)

Indeed, since the gimbal configuration is symmetrical and orthogonal in xy-axes, we applied the same control strategy to the angular displacement roll control (ϕ).

The algorithm control law $\upsilon_{\theta }$ is defined as

(25) $$\upsilon_{\phi }=-J_{x}\left[\frac{1}{\beta \gamma_{2}}sign^{2-\gamma_{2}}{\dot{\phi }}_{g}\left(1+\alpha \gamma_{1}{\left|\phi_{g}\right|}^{\gamma -1}\right)+\beta {\dot{\phi }}_{g}+K_{1}sign\left(s\right)+K_{2}s\right]$$

The control law $\upsilon_{\theta }$ for the roll ($\phi_{g}$) angle guarantees the finite-time convergence of the tracking error to zero.

### 3.3. Chattering Avoidance

It is well known that the implementation of the control approach based on sliding modes brings with it the disadvantage of the vibration effect present in the real-time experimentation of the sign function. In implemented control systems, it is important to ensure the performance of the system in the face of high working frequency and that the electronic controller can command in real time considering the electronic and mechanical wear of its actuators. However, in [8] the authors propose replacing the sign function with a high slope saturation function sign(·)≈sat(·) to solve this issue.

**Figure 5:**
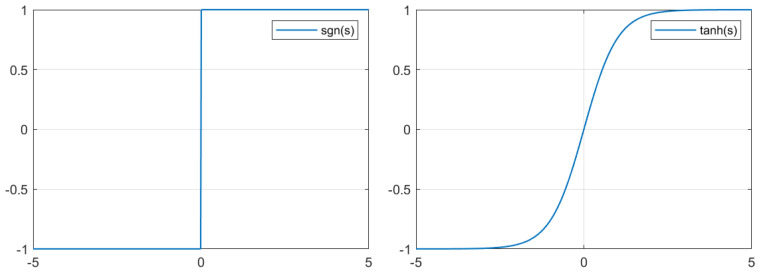
Signum function and hyperbolic tangent function.

In our approach, in order to avoid the chattering effect caused by the signum function in robust control algorithms used in the quadotor attitude and gimbal attitude, the approximation function sign(·)≈tanh(·) is used to smooth the signal. The signum function and hyperbolic tangent function are shown in Figure 5.

## 4. Materials and Methods

In this section, we will describe the different hardware parts of the embedded control system, namely, sensors, actuators, pilot communication, and the flight controller board that links them together. The total weight of the Unmanned Aerial Vehicle is 2.5 kg; the weight of the gimbal is clearly considered as the payload. The setup presented can be scaled to a smaller quadrotor aircraft by using a smaller gimbal or using servomotors instead of brushless motors for stabilization purposes. However, as in the case of Unmanned Aerial Vehicles, the gains must be adjusted in flight tests.

In order to guarantee the stability of the aerial vehicle for the development of the experiments through the optical flow module, an advanced autopilot called: Pixhawk 6X is used, which provides the corresponding measurements (attitude, position) for the high-performance control of our quadrotor aircraft.

Finally, the optical flow sensor presented in this work uses a stabilized gimbal to avoid the positive feedback produced when the vehicle tilts. The vehicle platform was built with the components shown in Table 1.

**Figure 6:**
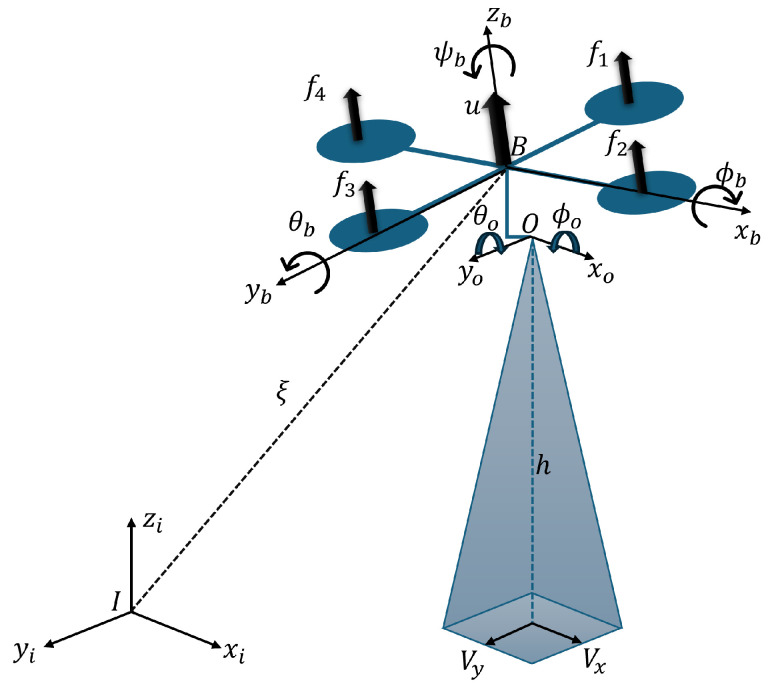
Navigation system: A quadrotor aircraft and a stabilized optical flow module.

Figure 6 shows the system diagram. A quadrotor aircraft and optical flow module are stabilized by gimbal, where $I=(x_{i},y_{i},z_{i})$ represents the inertial frame, $B=(x_{b},y_{b},z_{b})$ represents the body frame fixed to the center of mass of the quadrotor aircraft, and $O=(x_{o},y_{o},z_{o})$ is the frame fixed to the optical flow module. Finally, *h* is the altitude and $\left(V_{x},V_{y}\right)$ are the horizontal velocity estimations.

**Figure 7:**
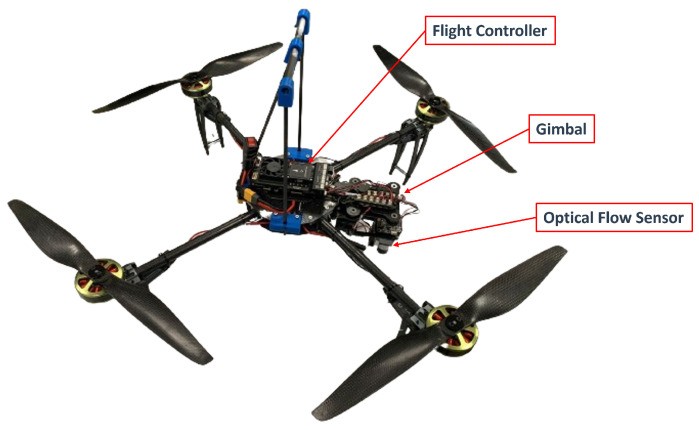
Experimental UAV platform.

The flight controller and custom PX4 firmware are used to control the quadrotor aircraft and stabilized gimbal. The modular flight controller has redundant embedded sensors (IMU’s), communication modules, and peripherals sensors. Figure 7 shows the experimental platform using an optical flow module stabilized by a gimbal. In this configuration, the experimental setup does not include GPS or MoCam.

The gimbal system configuration consists of two tilting joints around the $x_{o}$ and $y_{o}$ axis to control $\theta_{o}$ and $\phi_{o}$ close to zero. The gimbal mathematical model and robust control algorithm are important to ensure the performance through the torque stability of the motors. The gimbal robust control algorithm is implemented on a PX4 compatible controller board. In order to avoid the chattering effect caused by the signum function in robust control algorithms, we use the approximation function sign(·)≈tanh(·).

The improved optical flow module placed on the gimbal ensures that the estimation of the vertical position and the horizontal displacement velocity are reliably estimated regardless of changes in pitch-roll angles caused by the attitude disturbances or by the quadrotor aircraft displacement. Therefore, the optical flow angular velocities are close to zero $\omega_{x},\omega_{y}\approx 0$; Equation (5) can be rewritten as

(26) $$\begin{array}{c} V_{x}=\frac{T_{z}x-T_{x}f}{h}\\ V_{y}=\frac{T_{z}y-T_{y}f}{h} \end{array}$$

Therefore, the distance and optical flow sensor estimates can be used to control the position of the quadrotor aircraft considering the altitude h≈z and the horizontal displacement as

(27) $$\begin{array}{c} V_{x}\approx \dot{x}\\ V_{y}\approx \dot{y} \end{array}$$

## 5. Experimental Results

**Figure 8:**
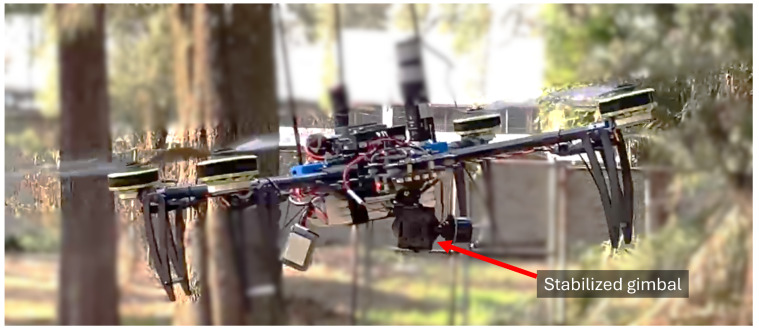
Stabilized gimbal in hover flight test with external perturbations.

To validate the proposed control strategies for robust attitude control implemented in the quadrotor aircraft and the stabilized gimbal, we will summarize the various real-time experimental tracking tests at different stages of development, see Figure 8.

The tracking performance can be observed as it reaches the desired position. The controller parameters were adjusted by trial and error until the best performance was obtained. The parameters used are shown in Table 2.

**Figure 9:**
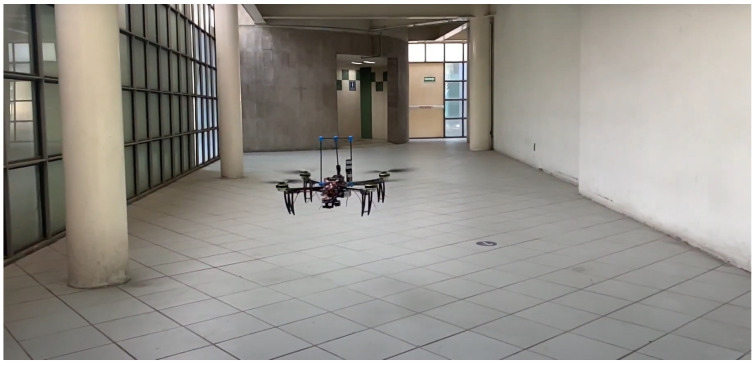
Indoor flight tracking control test with stabilized optical flow.

**Figure 10:**
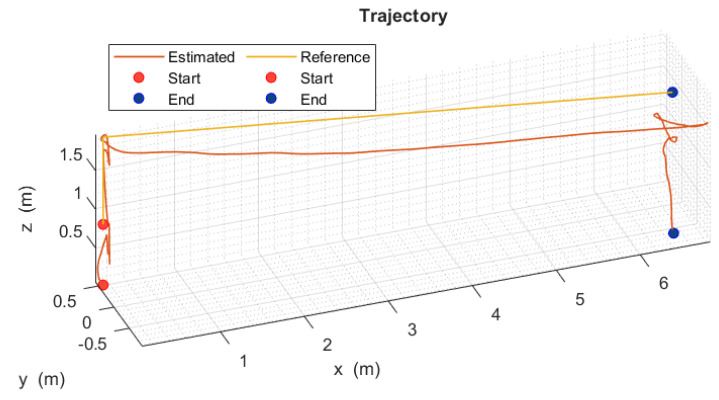
Three-dimensional trajectory tracking of the quadrotor aircraft (x,y,z).

Figure 9 shows an indoor flight tracking control test, and Figure 10 shows the trajectory in three dimensions of the quadrotor aircraft, which demonstrates the correct execution of the indoor flight mission using the optical flow sensor to track the programmed trajectory in the quadrotor aircraft at an altitude of 2 m and with a distance of 7 m.

In the following link, we share a video to show the disturbance rejection tests of the unmanned aerial system to demonstrate the quadrotor aircraft performance and the stabilized gimbal performance. Additionally, hover stability and a good performance in optical flow-based trajectory tracking control in a GPS-Denied environment are ensured.

→ https://youtu.be/SX1zxz5x9ko (accessed on 20 March 2024)

### 5.1. Quadrotor Aircraft Pitch and Roll Attitude (θ,ϕ)

**Figure 11:**
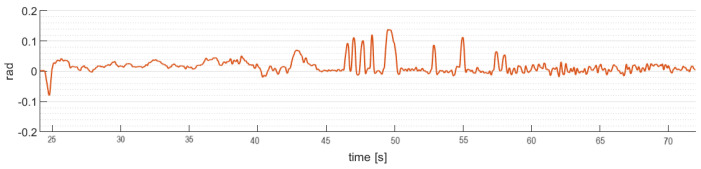
Quadrotor aircraft pitch angle (θ).

The attitude angles performance of the quadrotor aircraft during real time flight are presented below. Figure 11 shows the robust control law algorithm (20) behavior applied on the pitch angle stability of the quadrotor aircraft with disturbances added over the whole trajectory.

**Figure 12:**
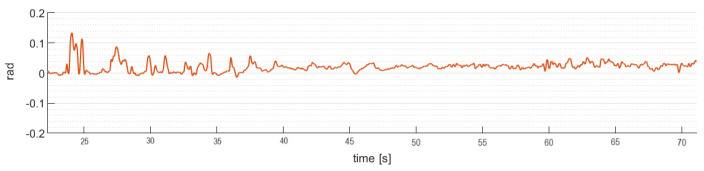
Quadrotor aircraft roll angle (ϕ).

Figure 12 shows the robust control law algorithm (20) behavior applied on the roll angle stability of the quadrotor aircraft, with disturbances added over the whole trajectory.

The quadrotor aircraft attitude angles (θ,ϕ) back to the reference in a short period of time, achieving normal operation after disturbances, with some oscillatory behavior around the horizontal reference values. Therefore, the results obtained are satisfactory.

### 5.2. Gimbal Pitch and Roll Attitude ($\theta_{g},\phi_{g}$)

The attitude angles performance of the stabilized gimbal during real time flight are presented below.

**Figure 13:**
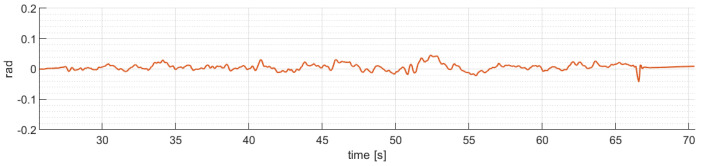
Gimbal pitch angle ($\theta_{g}$).

Figure 13 shows the robust control law algorithm (24) behavior applied on the pitch angle stability of the gimbal, with disturbances added over the fight.

**Figure 14:**
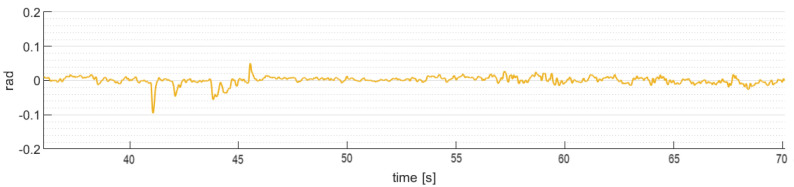
Gimbal roll angle ($\phi_{g}$).

Figure 14 shows the robust control law algorithm (25) behavior applied on the roll angle stability of the gimbal, with disturbances added over the fight.

The gimbal attitude angles ($\theta_{g},\phi_{g}$) back to the reference in a short period of time to normal operation after rejecting the angular disturbances induced by the quadrotor aircraft, with some oscillatory behavior around the horizontal reference values. Therefore, the results obtained are satisfactory.

### 5.3. Optical Flow Sensing ($z,V_{x},V_{y}$)

The altitude and displacement velocity sensing in real time flight are presented below.

**Figure 15:**
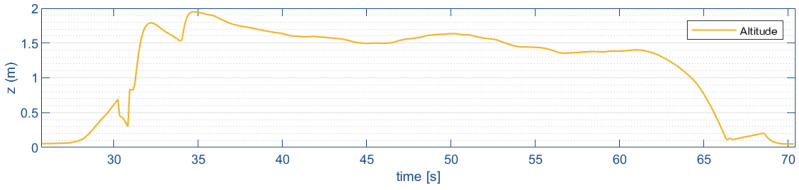
Vertical displacement (*z*).

Figure 15 shows the altitude estimate by the distance sensor to reach the desired altitude ($z_{d}=2$ m).

**Figure 16:**
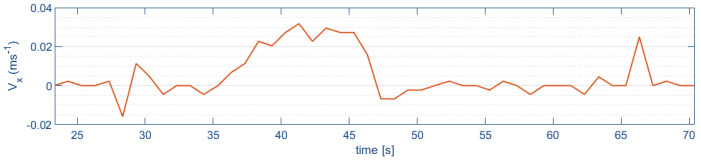
Lateral velocity ($V_{x}$).

Figure 16 shows the optical flow estimation of lateral velocity during indoor flight. An increase in speed of almost 0.04 m/s is observed during its lateral path of 7 m.

**Figure 17:**
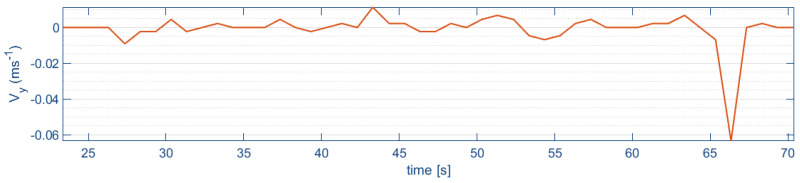
Longitudinal velocity ($V_{y}$).

Figure 17 shows the optical flow estimates of the lateral and longitudinal velocities, respectively, during the indoor flight. A lateral velocity of 0.01 m/s during a longitudinal path of 1 m is observed.

It is possible to observe a peak in the horizontal displacement velocity at the start and at the end of the flight. This occurs when the quadrotor aircraft does not take off or land with its full landing gear (due to disturbances, like the ground effect, or because the terrain is not flat and horizontal); the optical flow can detect a small horizontal displacement that increases with short focal length.

## 6. Discussion

There are many approaches around GPS-Denied navigation; some are mentioned in Section 1 of this work. The stabilized optical flow avoids the positive feedback of the velocity and position estimation. Regardless of the inclination rate and attitude change of the quadrotor, the stabilized optical flow will always maintain the downward orientation; this is the main goal of this work. However, a handicap of using a stabilized optical flow is the extra weight of the gimbal itself. Normally, the use of conventional optical flow sensors considers limitations in the translational velocity and the altitude of the sensor with respect to the ground as this altitude increases the estimation of the velocity, which is more sensitive to changes in sensor orientation. In our proposal, we are avoiding these attitude changes due to the stabilization of the optical flow. To carry out safe flight tests, it is required to know the operating limits of the sensors to produce reliable local estimates of altitude and horizontal velocity. For example, the maximum distance sensed by the optical flow module is 2 m, so we use a distance sensor with a longer operating range (40 m).

## 7. Conclusions

This paper has proposed a novel technique for navigating in a GPS-Denied environment using an optical flow module stabilized by a gimbal system. The proposed approach solves the problem of having a positive feedback in the pitch angle when the optical module is attached to the body of the aircraft. The technique consists of a control algorithm that keeps the optical module facing downwards so that the optical flow is independent of the pitch angle of the aircraft. We have successfully tested the proposed approach in an experimental platform in outdoor and indoor environments. The control algorithms of both the aircraft and gimbal were designed robustly to reduce the effect of external perturbations. The experiments showed that in spite of disturbances, the aircraft Euler angles remained close to the origin.

## Figures and Tables

**Table 1:** UAV components.

Carbon fiber airframe 550 mm	Pixhawk 6X Autopilot
Carbon fiber Propellers 1355 mm	Radio Control Receiver DSM
Outrunner Brushless Motors 335 KV	Telemetry Radio Sik
Brushless Electronic Speed Controllers 30 A	Rangefinder Lidar-Lite
LiPo Batery 5500 mAh 6S	Optical Flow PMW3901 Based Sensor

**Table 2:** System parameters.

Parameter	Value	Parameter	Value	Parameter	Value	Parameter	Value
$k_{p}$	0.36	$\delta_{1}$	1.45	$k_{b}$	0.86	$\gamma_{1}$	1.23
$k_{d}$	0.044	$\delta_{2}$	1.05	α	0.71	$\gamma_{2}$	0.95
$z_{d}$	2.0 m	$k_{1}$	0.54	β	1.61	$K_{1}$	0.68
$k_{a}$	0.084	$k_{2}$	0.33	ρ	1.23	$K_{2}$	1.33

## Data Availability

Dataset available on request from the authors.

## Abbreviations

The following abbreviations are used in this manuscript:

UAV	Unmanned Aerial Vehicle
VTOL	Vertical Take-Off and Landing
IMU	Inertial Measurement Unit
GPS	Global Position System
GRU	Gated Recurrent Unit
MARG	Magnetic, Angular Rate, and Gravity
FPV	First Person View
SMC	Sliding Mode Control
NTSMC	Nonsingular Terminal Sliding Mode Control
NTS	Nonsingular Terminal Sliding Surface
